# Sharon Operation under Spinal Anesthesia for Recurrent Sigmoid Volvulus: A Letter to the Editor

**DOI:** 10.70352/scrj.lte.26-0483

**Published:** 2026-10-06

**Authors:** Yu Kato, Hirotaka Honjo, Ryoma Sakamoto, Dan Liu, Hiroshi Kusanagi

**Affiliations:** 1 Department of Gastrointestinal Surgery, Kameda Medical Center, Kamogawa, Chiba, Japan; 2 Department of Anesthesiology, Kameda Medical Center, Kamogawa, Chiba, Japan

**Keywords:** Sharon operation, sigmoid volvulus, spinal anesthesia

## Abstract

The Sharon operation is useful for recurrent sigmoid volvulus in high-risk patients because of its minimal invasiveness and short operative time. We report the case of a patient with recurrent sigmoid volvulus who was at high risk during general anesthesia and successfully underwent the Sharon operation under spinal anesthesia. This case suggests that spinal anesthesia may be a feasible option for selected patients undergoing this procedure.

**To the Editor,**

We read with great interest the article by Inoue et al. on the Sharon operation; we were impressed by its practical value as a minimally invasive procedure for high-risk patients with recurrent sigmoid volvulus.^1)^ We would like to add an anesthetic perspective based on a patient in whom avoiding general anesthesia was considered important.

A 65-year-old man with cerebral palsy was referred to our hospital for definitive treatment of recurrent sigmoid volvulus. He had been hospitalized multiple times over the past 2 years. The patient presented with marked cervical and extremity contractures and stimulus-induced involuntary movements; however, his cognitive function was preserved. His American Society of Anesthesiologists physical status was class II. Preoperative assessment revealed respiratory muscle weakness secondary to cerebral palsy, raising concerns about the deterioration of his respiratory function, the potential need for reintubation, and the risk of postoperative aspiration.

Given these concerns, spinal anesthesia was chosen. It was performed at the L3–L4 interspace using a combination of 2.4 mL of 0.5% hyperbaric bupivacaine and 10 μg of intrathecal fentanyl. Given the preexisting respiratory muscle weakness, to minimize the risk of respiratory muscle impairment, the target sensory block level was set at Th 5. A bilateral sensory block to cold sensation was achieved at Th 5–6. The lateral decubitus position for spinal anesthesia was achieved without difficulty. To minimize involuntary movements operatively and provide analgesia, dexmedetomidine was continuously infused at 12–24 µg/h, with sedation titrated to a target Richmond Agitation–Sedation Scale score of −2 to 0. Ninety micrograms of fentanyl were administered intravenously and in intermittent boluses. Hypotension was managed with two 4-mg boluses of ephedrine. Surgery was performed in the supine position through a left lower quadrant pararectal incision measuring approximately 4.5 cm. Approximately 30 cm of the sigmoid colon was resected, and a stapled functional end-to-end anastomosis was created. The operation was completed in 38 min, and the anesthetic duration was 59 min. Oral intake was resumed on POD 1, and the patient was discharged on POD 7.

In our lean patient with a mobile, redundant sigmoid colon, the Sharon operation allowed for adequate access and manipulation through a small incision. General anesthesia with airway management using video laryngoscopy was considered as an alternative, but spinal anesthesia was selected to avoid postoperative respiratory and aspiration risks. Furthermore, dexmedetomidine sedation may have been beneficial owing to its modest analgesic properties and potential to prolong the analgesic effects of spinal anesthesia, while preserving spontaneous respiration and helping to control involuntary movements induced by surgical stimulation. However, neuraxial anesthesia alone may be associated with inadequate intraoperative pain control and reduced patient tolerance. Therefore, it should be performed only when the surgical and anesthetic teams are prepared for immediate conversion to general anesthesia.

This case suggests that the Sharon operation under spinal anesthesia may be a reasonable option for selected patients who are considered poor candidates for general anesthesia.
